# The mandibular incisive canal and its anatomical relationships: A cone beam computed tomography study

**DOI:** 10.4317/medoral.20644

**Published:** 2014-10-09

**Authors:** Patrícia Pereira-Maciel, Emerson Tavares-de-Sousa, Marcelo-Augusto Oliveira-Sales

**Affiliations:** 1 DDS, MSc. School of Dentistry, Federal University of Paraiba, Joao Pessoa-Brazil; 2 DDS, MSc. School of Dentistry, Federal University of Paraiba, Joao Pessoa-Brazil; 3 DDS, MSc, PhD. Department of Clinics and Social Dentistry, School of Dentistry, Federal University of Paraiba, Joao Pessoa-Brazil

## Abstract

**Background:**

To avoid postoperative injuries in the interforaminal region, presence of the Mandibular Incisive Canal (MIC), its extension and canal positioning in relation to the cortical bone and alveolar process were investigated by cone beam computed tomography (CBCT).

**Material and Methods:**

One hundred CBCT examinations obtained by means of the i-CAT CBCT imaging system were analyzed in multiple-plane views (axial, panoramic and cross-sectional) and three-dimensional representations were performed using iCAT CBCT software. The MIC was evaluated for its presence, measurement and proximity to the buccal and lingual plates, alveolar process and inferior border of the mandible.

**Results:**

The MIC was visible in all (100%) CBCT images. The mean length of MIC was 9.8 ± 3.8 mm. The distances between the canal and buccal plate, as well as between the canal and lingual plate of the alveolar bone were 4.62 ± 1.41 mm and 6.25 ± 2.03 mm, respectively. The distances from the canal to the alveolar process, and to the inferior border of the mandible were 10.25 ± 2.27 mm and 7.06 ± 2.95 mm, respectively.

**Conclusions:**

Due to the high prevalence of MIC, its variation in length and distance up to the cortical bone, suggested that preoperative radiographic evaluation of the MIC must be carried out case-by-case using CBCT, which could clearly show the three-dimensional structure and adjacent structure of the MIC.

**Key words:**Diagnosis, anatomy, cross-sectional, tomography.

## Introduction

The mandibular incisor nerve is described as the terminal branches of the inferior alveolar nerve that continues its intraosseous pathway into the mandibular anterior region, and provides innervations to the mandibular anterior teeth and canines (1). Some authors believe that the incisive nerve runs through the intramedullary spaces, and not within a bony canal, therefore, is not commonly detected by conventional radiography (1-3). Furthermore, anatomical studies using advanced imaging have shown strong evidence supporting the existence of the mandibular incisive canal (MIC) (3-9), located mesially to the mental foramen, smaller in diameter and less corticalized than mandibular canal containing the neurovascular bundle. (4-7,10).

During surgical procedures in the mandible, the mental interforaminal region is usually considered “a safe region” with minimal morbidity, however, it can exhibit important risks for anatomical and functional damage (5,11-14). The presence of the MIC is of significant interest, especially in patients who require surgical procedures in this region, including the insertion of endosseous implants, bone harvesting from the mental protuberance, genioplasty in orthognathic surgery, and with or without screw-retained plating after trauma in the anterior mandible (3-7,11,15,16). However, the presence of the MIC should not be underestimated during pre-surgical planning, and may cause postoperative sensory disturbances, edema, hematoma and lack of osseointegration of implants, pulp sensitivity changes, such as those that have been described in several reports (2,10,11,14-16).

Detailed preoperative study of the anatomical structures with Cone Beam Computed Tomography (TCCB) is crucial to success of the procedure (11,15), reducing the number of postoperative complications after selective procedures in the symphysis area. Several studies have shown that due to its excellent anatomic resolution, this exam is the best method for obtaining minimally invasive and accurate preoperative incisive canal measurements, because of their reproducibility and high degree of reliability (9,14,15,17,18), and have obtained similar results their anatomical studies (3,5-8,10,19).

Therefore, the aim of the present study was to quantify the presence of the Mandibular Incisive Canal, its extension and channel positioning in relation to the cortical bone and the alveolar process by cone beam computed tomography (CBCT).

## Material and Methods

This retrospective study included 100 randomly selected CBCT scans from patients of a private clinic, in accordance with the inclusion and exclusion criteria proposed ( Table 1). All images were taken by the same technologist, following a standardized protocol for patient positioning and exposure parameter setting. This research was approved by the ethics research committee of Lauro Wanderley University Hospital (169/10 CEP/HULW). The volunteers were included in the study after signing the Informed Consent Form.

**Table 1:**
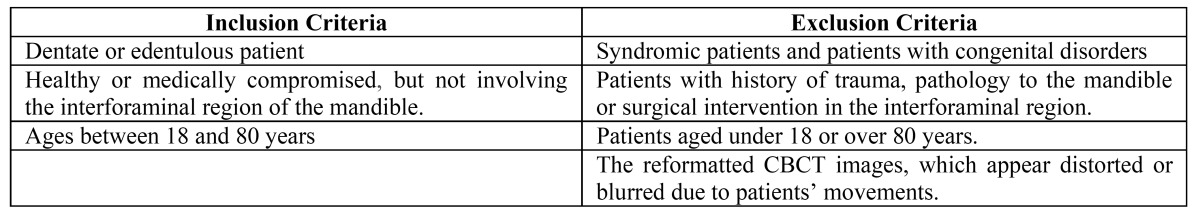
Inclusion and exclusion criteria.

**Figure 1:**
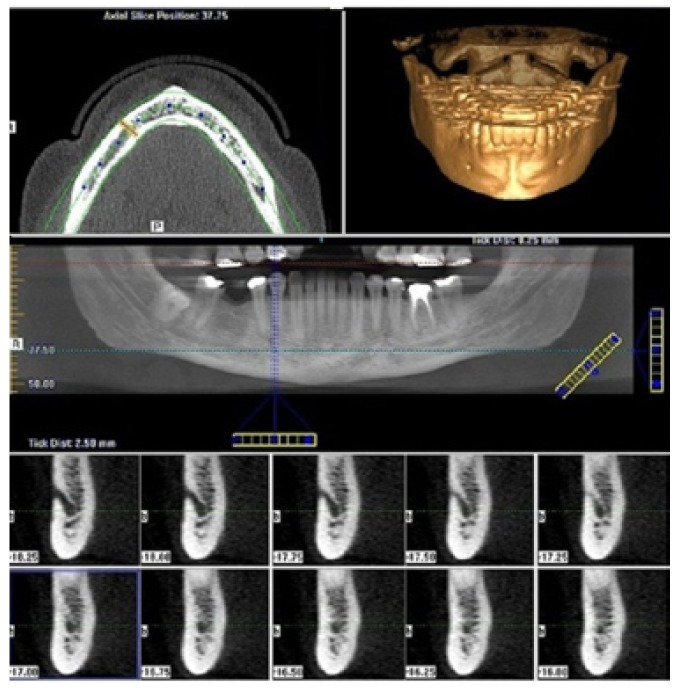
Multiple-plane reconstruction (panoramic and cross-sectional views). The course of the mandibular incisive canal (MIC) was located (A), from the closure of the mental foramen up to obliteration of the MIC (B-K).

The sample was acquired with the i-CAT® Cone-Beam 3D Dental Imaging System (Imaging Sciences International, Hatfield, PA, USA) using default parameters (120 kVp, 23.87 mAs, 6 cm field of view, 0.25 mm voxel size, 40s scan time, high-resolution bone filter). The DICOM data obtained were analyzed with a software program (i-CATVisionTMVisionQ version 1.8.1.10), reconstructed into multiple-plane views (axial, panoramic and cross-sectional views) and three-dimensional representations (Fig. 1).

The course of the mandibular incisive canal (MIC) was located, from the closure of the mental foramen up to obliteration of the MIC. The measurements were completed on cross sections perpendicular to a line parallel to the inferior border of the mandible. The two reference points included the incisive canal and inferior mandibular border. This plane passed through the inferior margin of the orbit and upper margin of each ear canal.

The course of MIC was assessed only in cross-sectional images and only if this structure was definitely visible. All Measurements were performed by two independent observers using the iCAT CBCT software program with a technical accuracy of 0.25mm and a maximum interobserver variability of 0.5mm. In particular, the following measurements were made:

a) After confirmed presence of the MIC, the visible length of the incisive canal, defined as the intrabony continuation of the mandibular canal mesial to the mental foramen was measured, given that the step was 0.25mm;

b) The distance from the MIC to the tooth apex or alveolar process;

c) The distance from the MIC to the labial bony surface.

d) The distance from the MIC to the lingual bony surface and 

e) The distance from the MIC Mandibular Border.

The prevalence in percentages was calculated for mandibular incisive canal. The comparison between the mean values was performed with the t-test for paired values (assuming equal and unequal variances). All statistical assessments were considered significant if *p*< 0.05. The software program used was SPSS for Windows (SPSS Inc., v17.0, Chicago, IL, USA).

## Results

The population consisted of 63 female and 37 male patients, aged between 20 and 80 years, with a mean age of 50.2 years, referred for several clinical reasons. The inter examiner and intraexaminer repeatability was tested and resulted in no statistically significant differences (*p* >0.05), indicating reliability.

For all CBCT images examined, it was possible to identify the incisive canal (100% were present bilaterally). The mean length of the incisive canal was 9.8 ± 3.8 mm, no statistically significant difference could be determined between the right and left sides (*p* =0.43) or with regard to gender (*p* =0.69) as shown in  Table 2.

**Table 2:**
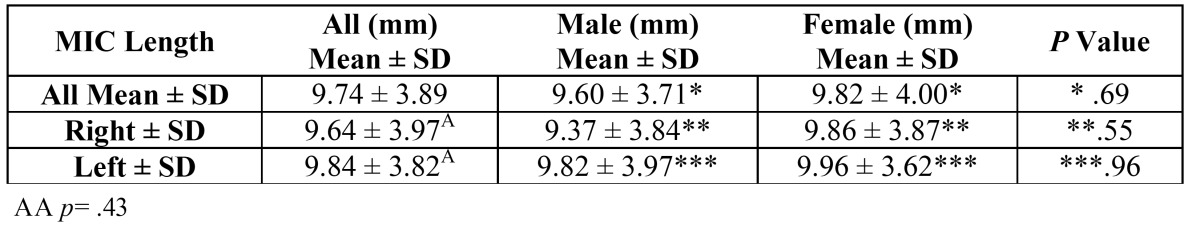
Measurement of MIC length.

Another finding was that the terminal part of the incisive canal showed significantly higher proximity to the buccal plate compared with the lingual plate (*p*<0.0001). The mean values measured were 4.62 ± 1.41 mm and 6.25 ± 2.03 mm, respectively. The terminal part of the incisive canal was in significantly closer proximity to the alveolar process, compared with the mandible border (*p*<0.0001), measuring 7.06 ± 2.95 mm and 10.25 ± 2.27 mm, respectively ( Table 3).

**Table 3:**
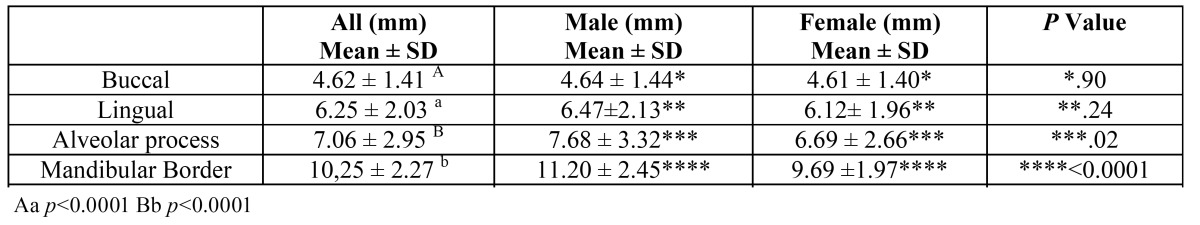
Values of the distance of the mandibular incisive canal (MIC) to various landmarks.

For measurements of the proximity to the buccal, lingual plate and alveolar process, no statistically significant difference could be determined between the right and left sides or with regard to gender, therefore, the data were analyzed as one unit (*p* >0.05). However, when the CBCT images were compared based on gender, the only statistical difference between the images (63 female and 37 male patients) was the measurements of proximity to the mandible border (*p* < 0.0001). The data indicated that this distance was shorter in females. In male and female patients, the mean values from the terminal part of the canal to the mandibular border was 11.20 ± 2.45 and 9.69 ±1.97, respectively, as shown in  Table 3.

## Discussion

Previous studies have investigated the mandibular incisive canal (MIC), but its existence is still widely debated, especially because it is considered an anatomical variation in this interforaminal region (4-8,10,20). This concept is due to the precarious detection of MIC by conventional radiography, a diagnostic method most frequently used up until recent times (5-8,10,21). Studies have reported that panoramic radiographs failed to detect the incisive canal (6,10,17,21-24). Thus, it has been suggested that this deficiency could be attributed to the smaller diameter and corticalization of the MIC, associated with the superposition of images observed in bidimensional radiographs (9,21,22).

Recent studies have reported that the mandibular incisive nerve has been found to be present in normal and atrophic mandibles (3,9,14,24), which justifies the inclusion of scans taken of dentate and partial edentulous patients. In this study was possible to identify the incisive canal in all CBCT scans, in reformatted cross-sectional images shown as a round radio lucent area within the mandibular trabecular bone, surrounded by a radiopaque rim representing the canal walls. These results were comparable with those reported by Al-Ani *et al*. (25).

According to the reports of Al-Ani *et al*. (25), the MIC was visible in all (100%) CBCT images, also using the original iCAT CBCT software program. Other authors have also found a high prevalence of MIC using CBCT, these with a variable visibility of 83-97.5%. In the study of Sokhn *et al*. (14), the incisive canal was identified in 97.5% of the images. Sahman *et al*. (17) reported the MIC was visible in 459 (94.4%) CBCT images. Apostolakis and Brown (26) identified the MIC in 93% of the cases. There was 91% visualization of the MIC by Makis *et al*. (7), Parnia *et al*. (20) found the MIC could be detected in 93.7% of the cases, whereas to Pires *et al*. (8), the canal was present in 83% das CBTC. Jacobs *et al*. (5) identified the MIC in 93% of spiral CT scans. Huang *et al*. (27) showed the presence of the MIC 78.75% (63 cases) of the CBCT scans.

The high prevalence of MIC found by means of the CT scan is comparable with direct measurement of cadaveric specimens (3,5-8,10), which can be considered a trusted method for the detection of this canal (5,8,9,14,19,23,25). In addition, Santos *et al*. (18) recently evaluated the reliability and reproducibility of measurements with CBCT, and demonstrated strong agreement between examiners. This could indicate that the methodology can serve as a standard for linear measurement analysis of the mandibular canal topography and adjacent osseous structures, with high accuracy and potential of providing unambiguous information for correct diagnosis.

Differences in the prevalence of this canal have been observed (3,5,7-9,17,20,25), when the canal is too small to be visualized on the CBCT (8), and when different systems have been used for obtaining tomographic images. These differed in sensitivity and slice thickness, because the smaller the voxel size used, the greater will be the detail of the reconstructed image (15). For this study, the voxel size used was 0.25 mm, less than all other studies (3,5,7,8,20,25,26). Another possible reason can be attributed to the fact that the MIC becomes smaller while progressing in the mesial direction to the mental foramen, to the most anterior part of the mandible, when it may be too small to be visualized on CBCT (4,5,8,27,28), but in the present study, it could be identified.

Although there are quantitative differences in the prevalence of MIC found in the literature, all agree that this prevalence is too high (5,7-9,17,20,24-26). Therefore, the concept of “a safe region” during surgical procedures in the interforaminal region should be questioned and a detailed study of the region must be performed during preoperative surgical planning.

For all CBCT images examined, the mean length of the incisive canal for the right side was 9.64 ± 3.97mm and for the left side was 9.84 ± 3.82mm. Despite the apparent difference between sides, there was no significant difference (*p* >0.05). There was also no significant difference between the genders. Similar lengths were obtained by Rosa *et al*. (9) and Apostolakis and Brown (26), measuring 9.11 ± 3.00 mm and 8.9 mm, respectively. Pires *et al*. (8) verified MIC lengths of 7.1 ±4 mm and 6.6 ± 3.7 mm for the right and left side, respectively. Another finding was that the MIC is in closer proximity to the buccal plate (4.62 ± 1.41mm) and alveolar process (7.06 ± 2.95), which is in agreement with other study (10). Although a tendency of MIC to approach the border of the mandibular and lingual wall has been noted, Apostolakis and Brown (26) and Rosa *et al*. (9) reported that MIC was also nearer to the buccal plate and alveolar process in its closest position, which is in agreement with the present investigation. Moreover, Rosa *et al*. (9) showed a downward path in only 51.3% of CBCT images. Furthermore, Huang *et al*. (27) observed that the mean diameter of MIC was 1.21 mm +/- 0.29 mm.

As regards gender, there was no significant difference when comparing the proximity of the MIC to the buccal and lingual walls and alveolar process, but the distance from MIC to the mandibular border was significantly lower for women than for men (*p*<0.0001). Al-Ani *et al*. (25) found that gender significantly affected all median distances and not only that of the mandibular border. This finding can be attributed to intrinsic differences existent in the bone structure of men and women. As women have a mandible smaller in dimension than that of men, if the MIC remained in the same position relative to the alveolar process for both genders, it would be closer to the edge of the mandible in women. However, the authors, did not measure the jaw size and jaw relationship to determine whether there really is a correlation (25).

Although the distances of the MIC from the bone plate seem to obey a pattern, the surgeon must be aware of the variable range of distribution of the MIC, as shown by Mraiwa *et al*. (6), Apostolakis & Brown (26) and Sokhn *et al*. (14), so that previously established default values may pose the risk of injury. The clinical significance of this study lies in the mapping of the incisive canal and its anatomical proximity during surgical procedures in order to avoid potential injury to the incisive mandibular nerve, a purpose safely achieved with the use of CBTC. Therefore, in order to determine the appropriate location of the MIC for each individual, this should be investigated on a case-by-case basis (2,8,14,20,25,26).

In conclusion, there is a high prevalence of the MIC for all CBCT images, with significant proximity of the terminal part of the incisive canal with to the buccal plate and the alveolar process. No statistically significant difference could be determined between the right and left sides or with regard to gender. The variation in length and distance up to the cortical bone suggested that preoperative radiographic evaluation of the MIC should be carried out case-by-case, using CBCT, which could clearly show the three-dimensional structure and adjacent structure of the MIC.
